# Giant Cell Arteritis Presenting as Bilateral Cotton Wool Spots

**DOI:** 10.7759/cureus.29804

**Published:** 2022-10-01

**Authors:** Lanxing Fu, Eoin P O'Sullivan

**Affiliations:** 1 Ophthalmology, King's College Hospital, London, GBR

**Keywords:** giant cell arteritis, cotton wool spots, granulomatous inflammatory vasculitis, polymyagia rheumatica, headache, scalp tenderness, jaw claudication

## Abstract

An 81-year-old Afro-Caribbean woman presented with a two-week history of a dull headache in her temples, jaw claudication especially when chewing food, and reduced vision in her eyes, more pronounced in the right eye. There was no past medical or family history of hypothyroidism or autoimmunity. On examination, the vision was counting fingers in the right eye and 6/36 in the left eye, best corrected. Dilated fundus examination revealed multiple peripapillary cotton wool spots in both eyes though more pronounced in the right. Her erythrocyte sedimentation rate (ESR) was 120 mm/h, and her C-reactive protein (CRP) level was 79 mg/L. A temporal artery ultrasound scan was undertaken immediately which demonstrated a halo sign around both temporal arteries and so a giant cell arteritis (GCA) diagnosis was made. The patient was commenced on daily high-dose IV methylprednisolone 1 g for three days and referred to the rheumatology team. Her vision improved to 1/60 right and 6/9 left eye best corrected at three days post-treatment. At 12 months after the initial presentation, her vision stabilized at 6/60 in the right and 6/6 with complete visual fields in the left eye. Cotton wool spots can be a sign of GCA. Their appearance with or without characteristic systemic symptoms should prompt urgent evaluation.

## Introduction

Giant cell arteritis (GCA) is a granulomatous vasculitis affecting medium and large-sized vessels and is the most common type of systemic vasculitis [1]. There is an increasing incidence with age, affecting between 15 and 25 per 100,000 over 50-year-olds [2]. Women have a greater preponderance, as do Caucasians and those of Scandinavian descent [3]. If untreated, GCA can lead to irreversible loss of sight and stroke and, therefore, is a medical emergency. The phenotype of GCA ranges from cranial to extracranial manifestations affecting the aorta, larger supra-aortic branches, and polymyalgia rheumatica (PMR). The most commonly affected cranial arteries are the temporal, ophthalmic, posterior ciliary, and vertebral. We present a case of GCA with typical and atypical features, which responded well to acute treatment and preserved vision.

## Case presentation

**Figure 1:**
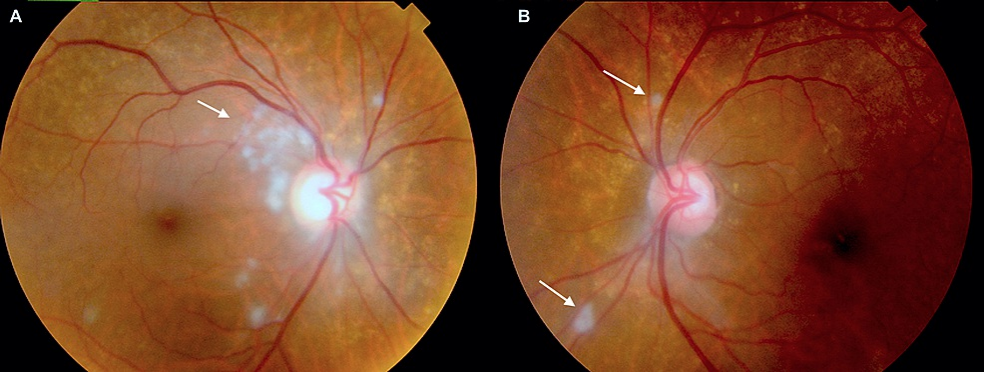
Fundus photo of the patient's right and left optic nerves. Panels A and B correspond to the patient's right and left retinae, demonstrating the peripapillary cotton wool spots on presentation (white arrows).

An 81-year-old Afro-Caribbean woman presented to the eye casualty service with a two-week history of a dull headache in her temples, jaw claudication when chewing food, and reduced vision in her eyes, more pronounced in the right eye. There was an absence of scalp tenderness, and shoulder or pelvic girdle pain. She did not have a fever, night sweats, weight loss, or a change in mood. The sole ocular history was elective cataract surgery three years ago with multifocal lens implants in both eyes. She had well-controlled asthma. There was no past medical or family history of hypothyroidism or autoimmunity. On examination, the vision was counting fingers in the right and 6/36 in the left eye, best corrected. Dilated fundus examination revealed multiple peripapillary cotton wool spots in both eyes, though more pronounced in the right (Figure 1). 

**Figure 2:**
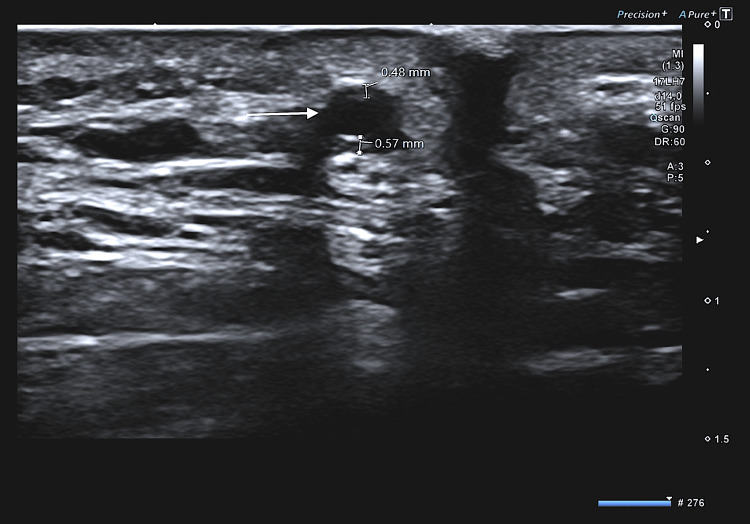
The halo sign (white arrow). Temporal artery ultrasound scan (Aplio i800 system; Canon Medical Systems, Tokyo, Japan) demonstrating the halo sign (white arrow). The thickened vessel walls measured 0.48 mm superiorly and 0.57 mm inferiorly in this image.

Her erythrocyte sedimentation rate (ESR) was 120 mm/h, and her C-reactive protein (CRP) level was 79 mg/L. A temporal artery ultrasound scan (Aplio i800 system; Canon Medical Systems, Tokyo, Japan) demonstrated a halo sign (Figure 2) around both temporal arteries, so a diagnosis of GCA was made. The patient was commenced on high-dose IV methylprednisolone 1 g for three days, reducing to oral prednisolone 60 mg OD until symptoms and laboratory results were within normal range. Her vision improved to 1/60 right and 6/9 left eye best corrected at three days post-treatment. A monthly tapering regimen was commenced starting from 60 mg OD and reducing to a maintenance dose of 10 mg OD over 6-8 months with serial monitoring of inflammatory markers. She also commenced on omeprazole 20 mg OD, alendronic acid 70 mg weekly, and calcium carbonate with colecalciferol 750 mg OD (Adcal-D3). She did not develop any glucocorticoid-related complications. 

**Figure 3:**
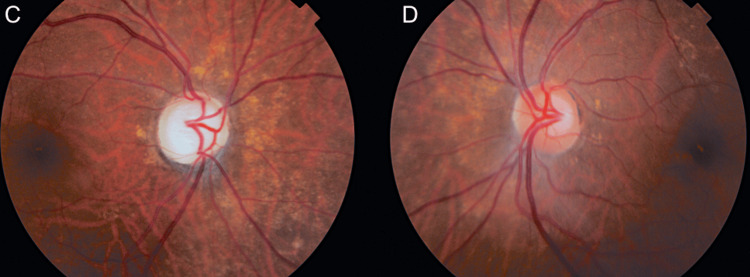
Resolution of cotton wool spots. Panels C and D are fundus photos of the patient at 12 months post-presentation, demonstrating the resolution of the cotton wool spots.

Her vision gradually reduced over a period of three to four months in the left eye and she was found to have left posterior capsular opacification of her intraocular lens. She subsequently underwent uneventful yttrium aluminum garnet (YAG) capsulotomy, to remove part of the opacified posterior capsule and ensure a clear visual axis. The cotton wool spots had completely resolved (Figure 3). Eleven months after the initial presentation, the patient stopped oral prednisolone for a few weeks and developed shoulder and pelvic girdle pain. Her inflammatory markers were found to be raised again (ESR 65 mm/h and CRP 29 mg/L compared with ESR 46 mm/h and CRP 16 mg/L one month earlier). She did not have any visual symptoms, and her vision measured 6/60 and 6/6 in the right and left eyes unaided with a complete left eye visual field. Unfortunately, the patient did not consent to a computed tomography-positron emission tomography (CT-PET) scan to review for possible large vessel involvement or an increase in the oral prednisolone dosage. She agreed to and tolerated the addition of weekly methotrexate 10 mg OD (Mondays) and folic acid 5 mg (Fridays).

## Discussion

The exact trigger for GCA is unknown, although strong associations have been demonstrated with genes of human leukocyte antigen (HLA) class II, plasminogen system, collagen synthesis, and proinflammatory cytokines [4]. Infection and inflammation are closely related, and infections were more common in GCA patients before diagnosis [4]. Herpes simplex virus, varicella-zoster, parvovirus B19, Epstein-Barr virus, chlamydia pneumonia, and mycoplasma pneumonia have been found in artery biopsy specimens [5-6].

Geo-environmental factors have been implicated in health, with a seasonal variation being found in biopsy-proven GCA in a 20-year retrospective study [7], however, a later meta-analysis that identified 22 studies did not confirm a seasonal onset for GCA [8].

There has been some debate on whether race can be a risk factor in GCA. In a recent retrospective cohort study comprising 586 patients who underwent temporal artery biopsy (TAB), there was no significant difference in biopsy-positive GCA cases between Caucasian and Afro-Caribbean patients. However, the proportion of Afro-Caribbean patients in the study was only 28.5% [9]. Similarly, in two other retrospective studies, the proportion of non-Caucasian patients was small [10-11]. Garrity et al. found no marked difference in the presenting feature of ophthalmic GCA (biopsy proven) between African-American (n = 32) and Caucasian patients (n = 84) in 10 institutions [12]. Some significant differences were found with African-American patients experiencing higher rates of headache, neck pain, anemia, and ocular pain [12]. However, the total sample of African-American biopsy-positive GCA patients was still only 32 [7].

Giant cell arteritis has a well-known association with PMR [13]. The presence of upper limb stiffness worse in the mornings, pelvic girdle pain, and constitutional symptoms (fever, weight loss, fatigue, malaise, chronic pain, etc.) are suggestive of PMR diagnosis necessitating further investigations.

The constellation of clinical signs and symptoms found in GCA can lead to diagnostic uncertainty and challenge. Classic clinical features are new-onset headache, scalp tenderness, jaw claudication, and systemic constitutional symptoms (fever, fatigue, anorexia, weight loss, PMR) [3]. Visual symptoms associated with GCA always necessitate a referral to ophthalmology and can be temporary or permanent. Cotton wool spots are due to localized axoplasmic debris accumulation and are associated with retinal ischemia [14]. They are found in diabetes, hypertensive retinopathy, rheumatological disease, and HIV. Vision loss occurs in up to 20% of GCA patients, primarily due to arteritic anterior ischaemic optic neuropathy, but also sometimes due to central retinal artery occlusion and posterior ischaemic optic neuropathy [14].

Clinical evaluation in combination with confirmatory testing is recommended to improve diagnostic accuracy [15]. The vascular ultrasound scan (US) in our case demonstrated the “halo sign” bilaterally, a non-compressible hypoechoic ring around the arterial lumen representing inflammation and subsequent edematous thickening of the arterial wall. Arida et al. described a sensitivity and specificity of 68% and 91% for the unilateral halo sign and a specificity of 100% for the bilateral halo sign [16]. The TABUL study found higher sensitivity with reduced specificity of US compared with the gold-standard TAB when combined with clinical evaluation [17]. Large-vessel involvement requires further imaging, e.g., computed tomography angiography (CTA), magnetic resonance angiography (MRA), and fluorodeoxyglucose-positron emission tomography (FDG-PET). Finally, there can be other causes of arterial vessel inflammation; hence clinical evaluation of the pre-test probability of GCA is still necessary. The significant advantage of the US is non-invasiveness and potentially time-saving than a TAB. Our patient was able to access the vascular US rapidly; fast-track pathways can reduce GCA-related morbidity and prevent complete visual loss.

The GCA management can be categorized into immediate, maintenance with or without adjuvants, and targeted therapy in refractory cases [15]. Often the patient presents with sight-threatening or with visual loss already present due to acute ocular ischemia. High-dose IV glucocorticoids, e.g., 1 g for three to five consecutive days, should be given if available, and oral prednisolone 60-100 mg if not [15]. Maintenance tapering regimens usually are between 12 and 18 months dependent on response, as 34%-62% of patients relapse [18]. Methotrexate can be combined with a glucocorticoid during the tapering stage to reduce the risk of glucocorticoid toxicity in high-risk patients [15, 19]. Tocilizumab is an interleukin-6 receptor antagonist and has been shown to delay the time to relapse and reduce the cumulative glucocorticoid dose in new-onset and relapsed GCA patients [20]. Ultrasound or TAB positivity is required to access tocilizumab in specific geographic regions, e.g. England. Therefore, confirmatory testing is essential.

## Conclusions

This patient presented with bilateral multiple cotton wool spots with raised inflammatory markers and disclosed no initial systemic symptoms. The utility of temporal artery ultrasound is demonstrated in this case without the need for invasive temporal artery biopsy. All patients would benefit from fast-track management pathways that involve a multi-disciplinary team, e.g. ophthalmology, rheumatology, internal medicine, neurology, and primary care. Immediate treatment and full evaluation are appropriate for patients presenting with signs and symptoms suspicious of GCA regardless of race.

## References

[1] Mollan SP, Grech O, O'Sullivan E, Mackie SL (2021). Practice points for ophthalmologists from the 2020 British Society for Rheumatology Giant Cell Arteritis guidelines. Eye (Lond).

[2] Mollan SP, Begaj I, Mackie S, O'Sullivan EP, Denniston AK (2015). Increase in admissions related to giant cell arteritis and polymyalgia rheumatica in the UK, 2002-13, without a decrease in associated sight loss: potential implications for service provision. Rheumatology (Oxford).

[3] Lyons HS, Quick V, Sinclair AJ, Nagaraju S, Mollan SP (2020). A new era for giant cell arteritis. Eye (Lond).

[4] Brault C, Riis AH, Mor A, Duhaut P, Thomsen RW (2018). Does low risk of infections as a marker of effective immunity predict increased risk of subsequent giant cell arteritis or polymyalgia rheumatica? A Danish population-based case-control study. Clin Epidemiol.

[5] Procop GW, Eng C, Clifford A (2017). Varicella zoster virus and large vessel vasculitis, the absence of an association. Pathog Immun.

[6] Alvarez-Lafuente R, Fernández-Gutiérrez B, Jover JA (2005). Human parvovirus B19, varicella zoster virus, and human herpes virus 6 in temporal artery biopsy specimens of patients with giant cell arteritis: analysis with quantitative real time polymerase chain reaction. Ann Rheum Dis.

[7] Gokoffski KK, Chatterjee A, Khaderi SK (2019). Seasonal incidence of biopsy-proven giant cell arteritis: a 20-year retrospective study of the University of California Davis Medical System. Clin Exp Rheumatol.

[8] Hysa E, Sobrero A, Camellino D (2020). A seasonal pattern in the onset of polymyalgia rheumatica and giant cell arteritis? A systematic review and meta-analysis. Semin Arthritis Rheum.

[9] Gruener AM, Poostchi A, Carey AR, Eberhart CG, Henderson AD, Chang JR, McCulley TJ (2019). Association of giant cell arteritis with race. JAMA Ophthalmol.

[10] Liu NH LaBree LD, Feldon SE, Rao NA (2001). The epidemiology of giant cell arteritis: a 12-year retrospective study. Ophthalmology.

[11] Tan N, Acheson J, Ali N (2019). Giant cell arteritis in patients of Indian Subcontinental descent in the UK. Eye (Lond).

[12] Garrity ST, Pistilli M, Vaphiades MS (2017). Ophthalmic presentation of giant cell arteritis in African-Americans. Eye (Lond).

[13] Smeeth L, Cook C, Hall AJ (2006). Incidence of diagnosed polymyalgia rheumatica and temporal arteritis in the United Kingdom, 1990-2001. Ann Rheum Dis.

[14] De Smit E, O'Sullivan E, Mackey DA, Hewitt AW (2016). Giant cell arteritis: ophthalmic manifestations of a systemic disease. Graefes Arch Clin Exp Ophthalmol.

[15] Mackie SL, Dejaco C, Appenzeller S (2020). British Society for Rheumatology guideline on diagnosis and treatment of giant cell arteritis. Rheumatology (Oxford).

[16] Arida A, Kyprianou M, Kanakis M, Sfikakis PP (2010). The diagnostic value of ultrasonography-derived edema of the temporal artery wall in giant cell arteritis: a second meta-analysis. BMC Musculoskelet Disord.

[17] Luqmani R, Lee E, Singh S (2016). The role of ultrasound compared to biopsy of temporal arteries in the diagnosis and treatment of giant cell arteritis (TABUL): a diagnostic accuracy and cost-effectiveness study. Health Technol Assess.

[18] Dejaco C, Brouwer E, Mason JC, Buttgereit F, Matteson EL, Dasgupta B (2017). Giant cell arteritis and polymyalgia rheumatica: current challenges and opportunities. Nat Rev Rheumatol.

[19] Best JH, Kong AM, Unizony S, Tran O, Michalska M (2019). Risk of potential glucocorticoid-related adverse events in patients with giant cell arteritis: results from a USA-based electronic health records database. Rheumatol Ther.

[20] Stone JH, Spotswood H, Unizony SH (2022). New-onset versus relapsing giant cell arteritis treated with tocilizumab: 3-year results from a randomized controlled trial and extension. Rheumatology (Oxford).

